# The mixed-meal tolerance test as an appetite assay: methodological and practical considerations

**DOI:** 10.1038/s41366-025-01866-7

**Published:** 2025-07-30

**Authors:** James A. King, Alice E. Thackray, Catherine Gibbons, Catia Martins, David R. Broom, David J. Stensel, Dimitris Papamargaritis, Frank Arsenyadis, Graham Finlayson, Gráinne Whelehan, Javier T. Gonzalez, John Blundell, Kristine Beaulieu, Lewis James, Lore Metz, Mark Hopkins, Masashi Miyashita, Scott A. Willis, Vicky Drapeau, David Thivel

**Affiliations:** 1 National Centre for Sport and Exercise Medicine, School of Sport, Exercise and Health Sciences, Loughborough University, Loughborough, UK; 2 NIHR Leicester Biomedical Research Centre, University Hospitals of Leicester NHS Trust and University of Leicester, Leicester, UK; 3 School of Psychology, Faculty of Medicine & Health, University of Leeds, Leeds, UK; 4 Department of Nutrition Sciences, The University of Alabama at Birmingham, Birmingham, AL USA; 5 Centre for Physical Activity, Sport and Exercise Sciences, Coventry University, Coventry, UK; 6 Faculty of Sport Sciences, Waseda University, Saitama, Japan; 7 Department of Sports Science & Physical Education, The Chinese University of Hong Kong, Hong Kong, China; 8 Diabetes Research Centre, University of Leicester, Leicester, UK; 9 Centre for Nutrition, Exercise and Metabolism, Department of Health, University of Bath, Bath, UK; 10 Clermont Auvergne University, EA 3533, Laboratory of the Metabolic Adaptations to Exercise under Physiological and Pathological Conditions (AME2P), CRNH, Clermont-Ferrand, France; 11 School of Food Science & Nutrition, Faculty of Environment, University of Leeds, Leeds, UK; 12 Department of Kinesiology, Faculty of Medicine, Université Laval, Quebec City, QC Canada

**Keywords:** Obesity, Translational research

## Abstract

Appetite control is a topic which attracts widespread interest given its importance to energy balance and obesity. In this research area, the mixed-meal tolerance test (MM-TT) has emerged as an ‘*appetite regulation assay’*, facilitating the dynamic assessment of appetite parameters (e.g. subjective appetite perceptions, appetite-related hormones, food reward) in response to an individual meal. The MM-TT is commonly employed in observational and experimental studies to examine population differences and intervention effects. Problematically, no practice standard exists for the MM-TT and protocols vary widely. This presents a challenge for researchers designing new MM-TTs and hampers the comparability of findings. Therefore, within this narrative review we sought to identify and discuss key methodological considerations inherent within a MM-TT. The scope of our review extends to evaluating participant familiarisation and methodological standardisation practices, test meal characteristics, appetite perception assessment, blood sampling techniques, measurement of appetite-related hormones and data handling/analysis. A checklist has been devised to summarise relevant methodological issues identified within this review. This checklist can be used as a tool by researchers to facilitate MM-TT design and promote greater standardisation/comparability between studies. This review highlights the need for broader standardisation of MM-TT procedures to support consistency across future research. Additional research is needed to strengthen the evidence base on which various recommendations are made, particularly relating to participant familiarisation and methodological standardisation practices. Additional scrutiny of less common outcomes employed in MM-TTs (not addressed here), such as diet-induced thermogenesis, gastric emptying and *ad libitum* energy intake, is also needed.

## Introduction

Appetite control is a topic which has risen in prominence, spurred largely (but not exclusively) by the need to tackle global obesity. This interest emanates from basic scientists keen to understand the psychobiological foundations of appetite [1, 2], as well as applied scientists focused on interventions (lifestyle, pharmaceutical, surgical). With the multi-dimensional nature of appetite, one of the key challenges in this field is the issue of measurement [3, 4]. That is, to identify which appetite constructs should be assessed and how their measurement is operationalised. This involves consideration of accuracy, precision and validity of measures [3] alongside a clear theoretical multi-disciplinary framework in which these constructs are considered.

**Fig. 1:**
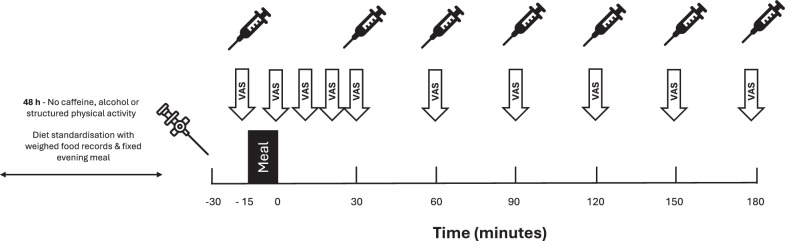
An example MM-TT. Intravenous cannulation is performed 30 min before the MM-TT for ‘habituation.’ Blood samples are collected for the analysis of appetite-related peptides. Each participant’s daily energy requirements are calculated based on their resting metabolic rate multiplied by their physical activity level (PAL). A balanced meal (50% carbohydrate, 35% fat, 15% protein) is consumed within 15 min after which postprandial measures begin. Note that a more frequent and longer duration blood sampling regimen may be desirable depending on the research question. VAS, 100 mm visual analogue scales for hunger, fullness, desire-to-eat & prospective food consumption.

Within appetite and energy balance science, the mixed-meal tolerance test (MM-TT) is a research method frequently employed as an ‘*appetite regulation assay*’. Practically, this method involves the repeated assessment of psychological and biological appetite parameters before and after a meal challenge (Fig. 1). The mixed (macronutrient) nature of this stimulus facilitates ecological validity and elicits holistic meal-related responses (cognitive and metabolic). The primary outcomes typically include appetite perceptions (i.e. visual analogues scales [VAS] for hunger, fullness, satisfaction & desire-to-eat) and circulating appetite-related hormone concentrations (e.g. ghrelin, peptide-YY [PYY], glucagon-like peptide-1 [GLP-1]). Additional outcomes also include food reward (e.g. Leeds Food Preference Questionnaire) [5], substrate oxidation, diet-induced thermogenesis and gastric emptying. The MM-TT is typically performed in a controlled laboratory environment with prior standardisation of dietary intake and lifestyle behaviours. A key principle of the MM-TT is that the dynamic assessment of appetite responses to a meal provides greater insight into appetite regulation than isolated, static (often fasted) assessments. The latter assessments typically capture a single time-point and may not reflect the complex, integrated physiological and psychological responses to eating that occur across the day. The protocol has theoretical parallels with the satiety cascade conceptual framework, originally proposed by Blundell et al. [6]. Specifically, the satiety cascade describes the timing and sequence of cognitive and physiological events that occur in response to food consumption which promote satiation (processes that bring an eating episode to an end) and satiety (processes that inhibit eating in the postprandial period). Herein, the MM-TT measures the post-ingestive consequences of eating food and therefore provides a measurement of satiety.

The MM-TT is commonly employed in observational (cross-sectional) studies to investigate differences between populations. For instance, comparisons between people with and without obesity [7, 8], or those who are physically active versus inactive [9]. The MM-TT is also used in experimental research to assess the effect of acute and chronic interventions. The latter include lifestyle interventions (e.g. exercise and diet) [10, 11], bariatric surgery [12] and obesity pharmacotherapy [13–15]. Additional applications include metabolic disease risk prediction in prospective studies [16, 17]. Problematically, there is no standard of practice for the MM-TT and protocols vary considerably across studies. This variation poses a challenge for researchers when designing new experiments and reduces comparability of findings. Methodological considerations include pre-test standardisation, test meal size and composition, test duration and measurement routine, assessment tools (e.g. questionnaires), blood sampling technique, analytes/biochemical assays, and data handling (statistical analysis) procedures.

This paper discusses key methodological elements about MM-TT design to support more informed decisions in human (predominantly adult) appetite research. The merits of different approaches are highlighted alongside recommendations for best practice. Because the MM-TT is a discrete technique, the scope of this review is limited to procedural elements that directly influence the MM-TT itself. Therefore, wider influences on energy homoeostasis or the longer-term control of appetite (e.g. body composition, physical activity, and energy expenditure) are not addressed. Equally, the use of MM-TTs to investigate other outcomes (e.g. metabolic and cardiovascular) is out of scope for this paper.

## Familiarisation and standardisation

### Familiarisation considerations

Ahead of MM-TTs, participants should be familiarised with research environments and phlebotomy procedures to attenuate anxiety and potential stress responses which may impact appetite [18, 19]. Once an intravenous cannula has been inserted, it is good practice to allow a ‘habituation period’ (15–30 min) before baseline assessments are made [18]. Participants should also be familiarised with study test meals (ideally consuming the foods) to attenuate novelty effects and VAS to ensure that participants understand scale constructs and descriptors.

### Standardisation considerations—meal presentation and consumption

Appetite and eating behaviour are influenced by many psychobiological factors. It is therefore important that the physical (i.e. appearance) and verbal presentation of the test meal are standardised. Specifically, participants should not be subject to external food cues during MM-TTs. This includes food odours [20], experimenter interaction, and exposure via the internet/mobile phones, videos and literature. Research has shown that even the size of cutlery and crockery require standardisation [21].

As appetite-related outcomes are sensitive to circadian rhythms, MM-TTs should begin at a fixed time that is replicated within and/or between participants. Typically, MM-TTs should begin early in the morning so that participants do not have to fast excessively. Morning tests—as opposed to afternoon or evening—also avoid logistical and cost issues associated with providing standardised breakfast and lunch meals (necessary for later MM-TTs). The time for test meal consumption must also be fixed (often 10–15 min) and standardised within participants so that meal duration does not confound outcomes. Moreover, for multi-item meals, the order of consumption should be standardised. Importantly, researchers must ensure that all of the test meal is consumed so that the stimulus is received as intended. To ensure this is achievable during the selected time-frame, pilot testing and familiarisation may be necessary. During the MM-TT, water consumption should be limited to a sensible standardised volume with the timing of consumption also standardised. The temperature and humidity of research environments should be comfortable (22 to 23 °C), monitored throughout the MM-TT, and replicated within and between participants. Environmental temperature is particularly important as it impacts appetite perceptions [22] and may arterialise blood, potentially impacting concentrations of circulating analytes.

### Standardisation considerations—pre-test diet

Standardisation of diet is commonplace before MM-TTs, particularly when repeated assessments are made within participants. This practice helps avoid pre-test energy and nutritional imbalance. To facilitate this, MM-TTs often commence in the morning with participants fasted, having not eaten since the previous evening. The timing and content of this final meal are commonly fixed. Weighed food records are often employed to facilitate dietary replication in the 24–72 h before MM-TTs. When financially and practically feasible, research teams may provide full standardisation diets. One study showed that 48 h of dietary standardisation was associated with lower variation in hunger responses to repeated test meals compared with no standardisation [23]. Without more evidence, a pre-test standardisation period of at least 48 h is recommended.

Hydration and alcohol intake are additional dietary considerations. Prolonged hypohydration may induce haemoconcentration and impact appetite perceptions [24]. Voluntary water intake is highly variable between individuals, but including water (and other fluid) intake as a recorded variable in the pre-testing food diaries can help to control this, at least within-participants, before testing. To help ensure euhydration, ~35–40 ml/kg/day of water (from food and drinks) in the days leading up to testing facilitates this, as well as consumption of a water bolus (~500 mL) in the hours before MM-TTs. However, consumption shortly before the MM-TT (i.e. <30 min) should be avoided, as acute water intake may suppress appetite and *ad libitum* energy intake [25]. Alcohol consumption may acutely affect appetite, eating behaviour, substrate metabolism, gastro-intestinal function and fluid balance [26]. As alcohol may specifically influence substrate metabolism for up to 24 h [27], we recommend prohibition of alcohol for 48 h before MM-TTs [23].

### Standardisation considerations—physical activity

Physical activity is commonly standardised in the 24–72 h before MM-TTs due to its influence on energy balance and acute appetite-modulating effects [28]. Additionally, participants should avoid physical exertion when travelling to research sites before MM-TTs. Accelerometry can be used to confirm physical activity standardisation in the days before MM-TTs (and during extended trials). While physical activity can be monitored in many ways, one potential method to facilitate standardisation is to pair a daily activity diary with any research-grade device capable of sensitively measuring steps per day. Device-measured steps provide an objective measure of overall physical activity volume and captures less formal or incidental movement. Concomitantly, an activity log facilitates the measurement of more formal activity, including that not captured by accelerometry (e.g. cycling) and the details of formal ‘training.’ Note that when studying active populations, researchers may wish to allow some activity in the days before MM-TTs—as the elimination of activity may represent an intervention. Recording and replication of activity is crucial in these scenarios. From a practical perspective, researchers may wish to limit any activity to that which is habitually undertaken by participants. Within the 24 h before the MM-TT, it may be prudent to request any activity to be completed early in the day to enable recovery. Without specific evidence to base a recommendation, a 48-h physical activity standardisation period may be prudent before MM-TTs. Some support for standardising physical activity 48 h before MM-TTs comes from evidence that exercise can improve insulin sensitivity within this time [29].

### Standardisation considerations—sleep

Sleep restriction impacts appetite perceptions, appetite-related hormones, food preferences and reward [30–32]. For instance, higher circulating ghrelin concentrations and hunger ratings were seen after just one night of sleep restriction [30]. Research participants should therefore be instructed to maintain adequate (7–9 h) and consistent sleep routines in the days before MM-TTs. A sleep diary (paper record of sleep onset and wake time) and/or an accelerometer capable of monitoring sleep variables—particularly night-time sleep onset and morning wake times—can facilitate this. Within individuals, standardisation of wake time is particularly important on the morning of MM-TTs [33].

### Standardisation considerations—smoking and caffeine

Smoking and caffeine intake are commonly controlled in appetite studies. As nicotine in tobacco suppresses appetite [34], tobacco/cigarette smokers are often excluded from appetite studies. If smokers are included, smoking routines should be standardised and recorded in the days before assessments. Participants should not be asked to stop smoking during this period, as withdrawal effects may affect study outcomes. On the day of MM-TTs, participants may smoke before MM-TTs (which must be replicated on successive assessments) but not during the procedure. Electronic cigarettes (e-cigarettes) and vapes also contain nicotine in combination with artificial flavourings and other chemicals which may alter the hedonic value and cravings for specific foods [35]. For these, the same standardisation procedures should be followed as with smoking [36, 37]. Marijuana (cannabis) may also influence appetite via orexigenic properties [38]. Unlike tobacco, only a small subset are daily or dependent users [39]; therefore, excluding these individuals may be appropriate unless central to the research question. In recreational users, although evidence is limited, withdrawals are uncommon and possible orexigenic effects are generally thought to persist directly and indirectly for up to 12 h [39–41]. Accordingly, abstinence for at least 12 h is recommended.

Caffeine may increase metabolic rate and suppress appetite and/or energy intake [42]. Therefore, highly caffeinated foods and beverages (especially coffee) are commonly prohibited 24–48 h before MM-TTs. However, the half-life of caffeine is ~4 h and withdrawal effects may be equally disruptive to behaviour. Consequently, moderation of intake, with standardisation across repeated tests, may be more appropriate in the prior 24–48 h; with abstinence on the day of the MM-TT. As the half-life of caffeine is ~73% longer (10.7 ± 3.0 h) in women taking oral contraceptives [43], researchers may wish to extend the abstinence period.

### Standardisation considerations—menstrual phase and contraceptives

Self-reported energy intake varies across the menstrual cycle in eumenorrheic women, being higher in the luteal versus follicular phase [44]. Cyclical changes in circulating sex hormones, impacting appetite perceptions, food cravings and food reward, may underpin this finding [45]. Standardisation of the menstrual phase is therefore important when investigating women in appetite-related research [46]. In experimental research, all women should be tested in the same menstrual cycle phase. Furthermore, it is important to consider which cycle phase women are tested in—especially if research involves investigating sex-based differences. Most often, comparisons are made to women in the follicular phase of the menstrual cycle, because oestrogen and progesterone levels are low, stable and more similar to men. Testing in the follicular phase therefore helps to limit any confounding influence of sex hormones on behaviour and metabolism. However, it must be recognised that women’s responses may differ if tested in the luteal phase, particularly as mood, cravings, water retention and gastrointestinal symptoms may differ. Therefore, testing in the luteal phase may provide a more ‘real-world’ comparison. In either case, unless women are tested in both phases, limitations of the approach taken must be explicitly considered. The variability in menstrual hormones between women means that within-subject designs are preferred over between-subject designs.

Verification of menstrual cycle phase is essential [46]. Whilst detailed monitoring of individual sex hormone profiles is the gold standard [46], this technique may be unfeasible. Other techniques that can be used in combination include cycle counting (forward and backward methods), ovulation monitoring (i.e. luteinising hormone tests), body temperature measurement, and wearable technology (i.e. multi-sensor tracking of physiological signals associated with cycle phase) [46, 47]. Given the difficultly in determining cycle phase, it is recommended that women with irregular, anovulatory or hormonally abnormal cycles should be identified in screening, analysed separately or excluded from research—depending on the research question at hand [46].

Whether individuals are taking oral contraceptives or are using other forms of hormonal contraception (e.g. intrauterine device) is an additional consideration in studies making comparisons between people (and should be documented). Whilst evidence is limited, oral contraceptive use may impact appetite-related neuro-peptide metabolism [48]. Consequently, it has been suggested that people taking contraception should form separate study groups and not be mixed with samples of participants who are not taking contraception [46, 47].

### Standardisation considerations—medications

A range of drug classes are known to influence appetite, energy intake, energy expenditure and/or body weight. These therapies may confound research when comparing different cohorts, or assessing intervention responses, particularly if medications change during a study. This is an important consideration when developing study inclusion/exclusion criteria and highlights the necessity to conduct thorough participant medication histories before enrolment. Whilst it is not possible to compile a complete list here, relevant common medications include certain anti-depressant and anti-anxiety medications [49], thyroid medications [50], corticosteroids [51] and therapies for obesity [52] (e.g. GLP-1-based medications) and diabetes (e.g. insulin, glitazones, metformin, SGLT2-inhibitors) [53].

## Meal stimulus

### Meal energy content

Deciding on the energy content of the meal stimulus is a challenging issue when designing MM-TTs. Initially, researchers must decide whether meals are provided as a fixed amount for all participants (akin to an oral glucose tolerance test in diabetes medicine), or whether energy content is ‘*scaled’* to a participant-level parameter. The former option is simple but can introduce between- and within-participant response variation, especially when the sample is physically diverse or has undergone significant intervention. The latter is more frequently chosen but raises further considerations about how the meal is scaled. Historically, meal energy content has most commonly been normalised to body mass, i.e. a set number of kilocalories per kilogram of body mass (akin to how various expert societies set out energy intake recommendations). This approach benefits from simplicity but is problematic when research participants vary substantially regarding adiposity e.g. if a study included people with and without obesity. In such cases, scaling to fat-free mass may be better. Alternatively, when feasible, meals can be scaled to resting metabolic rate (RMR) [54] or total daily energy expenditure (TDEE). The latter requires an additional estimate of movement-related energy expenditure (mainly necessary if participants vary significantly in their habitual activity levels), most precisely measured via doubly-labelled water techniques; but more economically and practically feasible via an accelerometer/inclinometer capable of estimating energy expenditure [55, 56]. Equally, movement-related energy expenditure can be estimated via self-report techniques, for example, established Physical Activity Levels (PAL, TDEE = RMR x PAL) [57]. In these cases, meal energy content is prescribed as a percentage of an individual’s measured or equation-derived (less optimal) values.

Within each scenario, researchers must determine the exact numerical values on which to scale. Practically, this calculation is made based on the test meal being scientifically and ecologically valid regarding size i.e. providing sufficient energy to elicit a meaningful appetite response (perceptually and metabolically) without invoking nausea or other adverse gastrointestinal symptoms, and similar to what participants would habitually eat (in a single meal context). When scaling test meals based on RMR and subsequent TDEE, meals are typically 20% to 30% of an individual’s daily energy requirements, to clearly differentiate a meal from snacking [58]. While standardising meal content across studies enhances comparability, there may be specific instances where non-standard scaling may be necessary. For example, individuals with reduced gastric capacity (e.g. post-bariatric surgery) or gastrointestinal conditions (e.g. gastroparesis, inflammatory bowel disease, functional dyspepsia) may not tolerate standard meal sizes, necessitating smaller/modified meals [59, 60]. This may also influence the MM-TT duration (typically 2–4 h) where the assessment period length may be modified given altered gastric emptying or other gastrointestinal dysfunction [59, 61]. Nevertheless, within a study, it is important all participants receive the same meal stimulus for internal validity, and test meals are trialled prior to the study for acceptability/tolerability.

### Macronutrient composition

Test meal macronutrient contribution is often determined as a secondary feature of the actual foods provided in a MM-TT. This is frequently determined based on palatability considerations for the local population (i.e. culturally-preferred foods) and practical considerations about meal provision (e.g. available preparation facilities). However, the composition of the test meal within a MM-TT is important as it impacts appetite and metabolic responses postprandially. For example, on a per kilocalorie basis, protein generates the strongest satiety response, and fat the weakest [62]. Moreover, the magnitude of response of appetite-related peptides to meals varies depending on the macronutrient stimulus [63]. Beyond this, qualitative differences in the types of carbohydrate, protein and fat, or the confounding effect of soluble/insoluble fibre, may further impact postprandial appetite and metabolic responses. For example, offering equal amounts of dairy-based casein versus whey protein, which have known differences in amino acid profile and postprandial metabolic response, have differing postprandial VAS scores for satiety and fullness [64]. Similarly, isoenergetic meals with higher or lower ratios of simple versus complex carbohydrates also provoke different postprandial effects on hunger, satiety and metabolic parameters [65]. Additionally, polyunsaturated and saturated fatty acids differentially impact post-meal appetite-related hormone responses [66]. Therefore, differences in test meal composition directly contribute to variability in meal responses between studies, which hampers comparability of research findings.

When considering MM-TTs using whole foods, it is practically impossible to recommend a universal standardised test meal to use given international differences in habitual dietary intake, food availability and food preferences. However, most studies in the literature have opted for ‘*balanced’* test meals containing ~50% carbohydrate, ~35% fat, and ~15% protein. This likely reflects Western food preferences and the predominance of research studies from high-income Western nations in the scientific literature. While specific food items selected may differ between studies based on cultural norms or studying populations with specific dietary requirements (e.g. gluten intolerance), researchers may still wish to opt for a ‘*balanced*’ profile. Such a meal profile is broadly consistent with international recommendations for healthy diets and ensures that each macronutrient is sufficiently represented within the meal stimulus. In the absence of a popular alternative, researchers may wish to replicate this meal profile to facilitate comparability.

### Physical state

The physical state of the test meal is another consideration for researchers undertaking MM-TTs. Whilst most studies base their meal stimulus on whole foods, oral nutritional supplements, i.e. nutritionally-complete, ready-to-drink liquid meals (often milkshakes or juices), can also be used [13]. Whilst there are merits in each approach, the postprandial appetitive response is qualitatively different. Importantly, whilst metabolic and hormonal responses are comparable, liquid meals produce weaker satiety responses compared with solid meals [67]. Nonetheless, oral nutritional supplements benefit from their ease of preparation (reducing human error introduced while weighing and preparing food), homogeneity in content, and validated nutritional profile (including the ability to exclude allergens). Moreover, the nature of oral nutritional supplements facilitates swift consumption, potentially reducing variability in meal duration between people, and allows for blinding where relevant for certain research questions. Equally, participants’ lack of familiarity with oral nutritional supplements attenuates the impact of prior interactions/preferences on satiation and satiety, which may be particularly important in cross-sectional studies involving single assessments. Alternatively, meals based on whole foods have greater ecological validity i.e. better reflect real-life. Notably, the requirement to chew whole foods influences a range of psychobiological (appetitive) responses [68], which are part of the normal process of eating. Overall, the approach taken will be influenced by the research question at hand alongside practical considerations.

## Appetite scales/questionnaires

Appetite-related self-reports include several items intended to capture, over a given period, specific subjective sensations or perceived motivations to eat. These include general feelings of hunger/fullness, desire to eat (in general or specific food types), and prospective judgements about the quantity of food or specific food types that could or would be eaten. Appetite-related VAS have been used extensively in studies examining motivation to eat in relation to food preloads, test meals, pharmacotherapies and medical devices, as well as dietary, exercise, and sleep interventions.

The appetite VAS typically include a series of 100-mm horizontal lines representing continua anchored at either end by the absence or extreme presence of a subjective appetite-related state [69]. Although the 100-mm VAS length is most commonly used, the 150-mm VAS is also valid and leads to similar results [70]. Therefore, appetite VAS scores can be obtained from 100-mm and 150-mm length scales both before and in response to a meal [70]. The participant indicates the point along each line that best represents how they feel at that moment.

The most commonly-used version of this questionnaire includes appetite dimensions specific to hunger, fullness, desire-to-eat, and prospective food consumption. Hunger and desire-to-eat are thought to be indices of a construct that reflect subjective hunger, while fullness and prospective food consumption are considered to reflect satiety. It is important that the differences between the four items are explained to participants prior to being asked to complete VAS ratings. A variant of the appetite VAS used in some European countries also adds a question on satiety [71]. Additionally, desire-to-eat can be expanded to desire-to-eat savoury/sweet foods, or other food types, as well as feelings of thirst, nausea and bloatedness. Description and specific questions pertaining to the five most commonly-used VAS are presented in Table 1.

**Table 1:** Description of perceived motivation to eat.

Dimension	Description	Question	Appetite state
Hunger	Captures immediate motivation to eat.	How hungry are you?	Ranging from “Not hungry at all” to “Extremely hungry”
Fullness	Reflects the feeling of being full or post-meal satiation.	How full are you?	Ranging from “Not full at all” to “Extremely full”
Desire-to-eat	Measures the general or specific DTE.	How strong is your desire to eat?	Ranging from “No desire to eat” to “Strong desire to eat”
Prospective food consumption	Measures the anticipated quantity of food that could be eaten.	What amount of food could you eat?	Ranging from “Nothing” to “A very large amount”
Satiety	Measures the feeling of being satiated or satisfied.	How satiated are you?	Ranging from “Not at all satiated” to “Extremely satiated”

*DTE* desire-to-eat, *PFC* prospective food consumption.

To capture the immediate peak/nadir within meal responses, each appetite sensation should be assessed separately with measurements taken soon after arrival, immediately before and after the test meal, and at the following time-points: 10, 20, 30, 60, 90, 120, 150, and 180 min post-MM-TT [3]. Researchers may wish to perform additional assessments beyond this point, for example, if blood analytes are measured over a longer duration. Appetite VAS should be assessed before blood samples are obtained to reduce any influence of potential uncomfortable feelings during the insertion of a cannula or sample collection. Electronic devices can be accepted as a valid method for measuring appetite but should not be used interchangeably with paper and pen [72]. While data collected using the paper and pen method may not differ from that collected electronically, the former is more time-consuming as individual lines are measured manually and inputted into a spreadsheet, potentially introducing human error. When combined with the standardisation protocol outlined in this article, appetite sensations have been shown to be both valid and reproducible [23, 73]. The validity of appetite sensations as a predictor of energy intake has been demonstrated in numerous studies [74].

### Presentation of appetite VAS scores

In addition to analysing single time-points or graphical curves, researchers have developed various formulae and statistical approaches to enhance the utility and sensitivity of the basic VAS scores. Participants’ ratings for individual VAS (hunger, fullness, desire-to-eat, and prospective food consumption) are often analysed separately, but may be combined to summarise ‘motivation to eat’ [75]. As such, when analysing VAS, it might be advisable to calculate a composite appetite score (CAS) based on all of the scale items, unless there is a specific hypothesis relating to individual items. Table 2 provides an in-depth summary of the most commonly presented VAS outcomes and their calculation, appropriate use, and example applications. Additional elaboration is given in the data handling section of this paper.

**Table 2:** Summary of outcomes, formulae and statistical approaches based on the appetite visual analogue scales (VAS).

Method	Description	Calculation	Reliability/validity	Discussion	Examples of application
Single time points	Measures appetite at specific moments such as fasting or pre-meal	Individual appetite VAS scores at distinct time points for each appetite sensation	Depends on timing and standardisation and does not reflect satiety response [3]. Often leads to many comparisons, increasing type 1 error.	Provides snapshots of appetite states for comparison. Could be used to compare pre-meal appetite differences in MM-TT.	Fasting studies, pre- and post-meal evaluations studies [23].
Multiple time points	Measures appetite over periods of time	Raw data or changes from baseline (baseline subtraction) for each appetite sensation	Method validated in many studies [3]. Baseline variables should be considered in the statistical analyses.	Changes from baseline are often preferred in MM-TT to account for pre-meal appetite differences and to isolate the postprandial response. However, both approaches can be used depending on the study objective and the nature of the intervention.	Used in studies to compare conditions [141].
Rate of recovery	Measures the time it takes for appetite levels to return to baseline after a meal or intervention	Change in appetite scores over time (e.g., slope of recovery curve) for each individual appetite sensation	Validity and reliability in specific contexts [142].	Useful in assessing the duration of satiety or hunger suppression	Used in food preloads, meal composition, and pharmacological agent studies [143, 144].
Early versus late satiety	Differentiates satiety responses at early versus later stages of the postprandial period	Comparison of appetite scores at early (mean of 0–60 min period) and late (mean of 60–180 min period) postprandial time points for each appetite sensation.	Method from Gibbons et al. [141], validated in satiety studies.	Highlights differences in satiety dynamics across time.	Used in meal structure and nutrient timing studies [141, 145]
Area Under the Curve (AUC)	Summarises the overall appetite response over a specified period	Calculated using trapezoidal method: the mean scores of pairs of adjacent time points are multiplied by the time interval between the sampling points [125]. Total AUC includes the total area between the origin line (zero) and response curve; incremental AUC is restricted to areas above a nominal threshold (most often baseline) [126, 128, 146]	Trapezoidal method is widely used and validated [126, 146].	Provides a comprehensive measure of appetite over time representative of the magnitude and duration of response. Incremental AUC may be more appropriate if baseline differences are evident and/or the response to the meal is the primary interest.	Applied in preload and intervention studies [72].
Composite Appetite Score (CAS)	Combines multiple appetite measures (e.g., hunger, fullness, desire-to-eat, prospective food consumption) into a single index	Average or weighted combination of individual appetite scores (usually four dimension – ([hunger + DTE + PFC + (100−fullness)]/4)	Method validated in multiple contexts [147].	Enhances sensitivity and utility of appetite data.	Studies requiring a global appetite measure [148, 149].
Satiety Quotient (SQ)	Evaluates the satiety efficiency in response to food intake.	Change in appetite scores divided by energy content of the meal [Mean 1 h post appetite scores-pre/kcal MM-TT*100]. Can be used for an individual appetite sensation or average appetite scores (e.g. hunger, fullness, DTE and PFC).	Method from Green et al. [150], validated against reported and measured energy intakes [151].	Links subjective appetite to objective energy intake, emphasising food quality and quantity.	Used to document the strength of satiety related to biology of the individual satiety efficiency research, preload studies, and portion control interventions [152, 153].

### Additional considerations

Appetite VAS are cheap, easy to use, and simple to interpret. These scales demonstrate good within-subject reliability and validity [23] and broadly predict food intake [74]. Values also vary under conditions where they would be expected to change (e.g. in response to nutrient loads and anorectic drugs) [74]. However, the between-participant variability can be large, meaning VAS are more suitable for within-subject comparisons than between-subject comparisons. Robust familiarisation with VAS, and strict standardisation of procedures, before and during the MM-TT, limit the impact of confounding factors on VAS data. Importantly, VAS should not be used directly as a proxy for quantitative variables such as energy or food intake as they do not always predict these outcomes perfectly. As such, if energy or food intake is a primary outcome of the study, an *ad libitum* test meal should be used at the end of the MM-TT period.

The Three-Factor Eating Questionnaire (TFEQ) [76] and the Dutch Eating Behaviour Questionnaire (DEBQ) [77] are psychometric tools used in appetite studies to assess eating behaviours and attitudes towards food. As the constructs within these questionnaires (TFEQ: dietary restraint, disinhibition, hunger; DEBQ: emotional, external and restrained eating) can influence research outcomes, these tools are often employed during pre-screening to identify, screen and stratify individuals exhibiting extreme values. Additional eating behaviours or eating traits may also influence MM-TT outcomes, including but not limited to: food cravings, eating disorders, body image disturbance, mood/affective states, impulsivity/reward states, mindful eating and interoceptive awareness. Researchers should therefore use their judgement to determine whether other questionnaires should be employed before an MM-TT, to screen in/out participants or to retrospectively permit statistical adjustment and sensitivity analyses.

Although VAS are widely used in appetite research, it is important to acknowledge that they may be less suitable in certain situations. For example, difficulties may arise for individuals who have trouble understanding abstract concepts such as rating feelings on a scale, including those with visual impairments, cognitive impairments, low literacy levels, or language barriers. Young children (<7 years) are one prominent example, where more bespoke methods (e.g. picture-based scales) may be needed to assess appetite [78, 79].

## Blood sampling

Sampling blood before, and frequently after ingesting a MM-TT provides insight into metabolic and hormonal responses. This may enhance understanding of factors influencing appetite-related outcomes. The method of blood sampling (e.g. location, frequency and posture), can impact concentrations of circulating analytes and requires consideration.

### Location of blood sampling

Location choice will depend on the research question and resources available. Generally, in physiological research, the most common locations of blood sampling are capillary beds (e.g. sampling from a fingertip, earlobe, or heel), and veins within the antecubital fossa of the forearm, providing mixed-venous blood. Capillary sampling is inexpensive and less technically challenging but limited by sample volume, making venous blood the common choice when several analytes are measured.

**Fig. 2:**
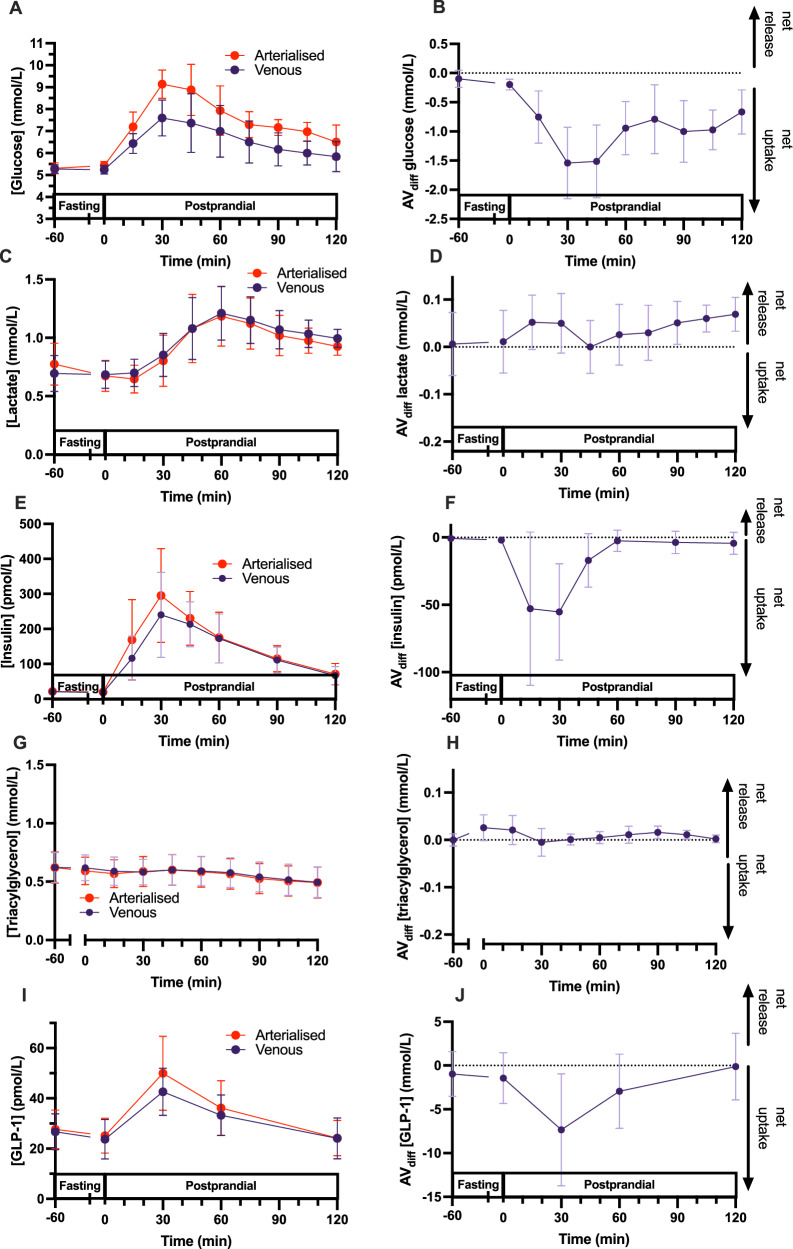
Metabolite responses to a standard oral glucose tolerance test at different sampling locations. Plasma glucose (**A**, **B**), lactate (**C**, **D**), insulin (**E**, **F**), triacylglycerol (**G**, **H**), and glucagon-like peptide 1 (GLP-1; **I**, **J**) concentrations in arterialised and mixed venous blood (**A**, **C**, **E**, **G**, **I**) and net forearm uptake/release (**B**, **D**, **F**, **H**, **J**) before and after ingestion of 75 g glucose. Data redrawn from 10.1017/S0007114517001362 and 10.1113/EP087118.

A more technically challenging method is sampling arterial blood, which provides the best indication of peripheral analyte availability, since arterial blood is delivered to most tissues. As tissue metabolism can alter the composition of the blood via net uptake, release or interconversion of an analyte, sampling non-arterial blood can misrepresent the peripheral availability of an analyte under some circumstances. The extent to which the sample location makes a difference to inferences drawn depends on factors such as the net tissue flux of the analyte and tissue blood flow. For example, after oral ingestion of a 75 g glucose load, there is a net uptake of glucose, insulin and GLP-1, and net release of lactate by the forearm, whereas the net forearm flux of triacylglycerol remains neutral (Fig. 2) [80–82]. Accordingly, for the most accurate representation of postprandial secretion and availability of analytes for the periphery, arterial or arterialised blood may be preferable to venous blood for many metabolites and hormones.

If arterial blood is most appropriate for the research question but is not practically feasible, then *arterialised* venous blood and capillary samples can replace arterial sampling for some analytes but not necessarily appetite related peptides. To obtain *arterialised* venous blood, retrograde cannulation of a pre-heated dorsal hand is needed and measuring oxygen saturation in blood samples can confirm arterialisation. The arterialisation method has been shown to provide samples representative of arterial blood under various conditions for some analytes; including, glucose, lactate, insulin, non-esterified fatty acids (NEFA), and beta-hydroxybutyrate [83–85]. However, it has been suggested that the method may affect some counter-regulatory hormone responses to hypoglycaemia (i.e., glucagon secretion and/or clearance) [86]. It is worth noting that in many circumstances, concentrations of analytes (e.g., glucose) within capillary samples are closer to arterial/arterialised than to venous samples. Therefore, capillary sampling can be an alternative to arterial/arterialised sampling if the required sample volume for analysis is relatively small. To our knowledge, representation has not been demonstrated in appetite-related peptides so further research is necessary to demonstrate if this is the case. Researchers are therefore encouraged to follow blood sampling guidelines in assay instructions meticulously.

### Frequency and duration of blood sampling

The appropriate frequency and duration of blood sampling depends on the research question, resources, and study population, in addition to other aspects such as the size and composition of the MM-TT. Frequency of blood sampling should be sufficient to capture the required resolution of a postprandial response, ideally detecting a peak or nadir concentration of an analyte. Duration of sampling should ideally aim to capture the full postprandial response (with the return to fasting/basal concentrations) and at least capture a peak/nadir concentration.

**Fig. 3:**
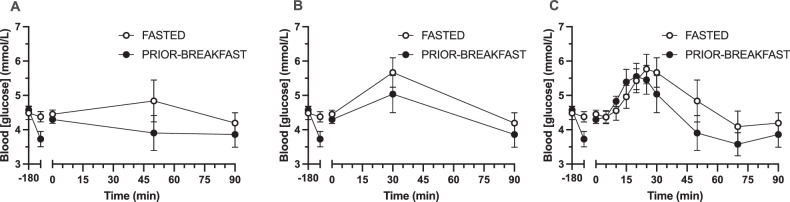
Circulating blood glucose profiles in response to fasting and breakfast consumption at different sampling intervals. Panels show circulating blood glucose concentrations with blood sampling conducted at 50 min (**A**), 30 min (**B**) and at 5-min intervals (**C**). Data taken from: 10.1017/S0007114512005582.

In insulin-sensitive populations, glucose, insulin and NEFA concentrations typically achieve peak/nadir concentrations within 60 min and return to fasting concentrations within 120 min [87, 88]. In contrast, circulating ghrelin, gastric inhibitory polypeptide (GIP), GLP-1, and PYY concentrations may take longer than 180 min to return to basal [88–90]. The potential impact of sampling frequency on assessing a postprandial response is illustrated in Fig. 3, using data from published work [91]. An inference based on infrequent sampling would be that peak glucose concentrations would be achieved at 50 min after ingestion, and that prior breakfast consumption would result in no subsequent postprandial rise in glucose concentrations (Fig. 3A). Using the same data with increasing sampling frequency, an additional time-point of 30 min postprandially would result in an inference that peak glucose concentrations are achieved at 30 min in both conditions (Fig. 3B), and with 5-min sampling frequency, there is evidence of a difference in the time of peak glucose concentrations between conditions (20 vs. 25 min; Fig. 3C). A summary of recommendations regarding sampling location, frequency and duration can be found in Table 3, although researchers may have valid reasons for alternatives to these recommendations.

**Table 3:** Recommended priority sampling location, frequency and duration for various analytes.

Analyte	Recommended sampling location	Ideal sampling frequency (min)	Ideal sampling duration (min)
CCK [145]	Arterialised, Capillary, or Mixed Venous.^a^	≤15 min	≥180 min
Glucagon [154]	Arterialised, Capillary, or Mixed Venous.^a^	≤15 min	≥240 min
Ghrelin	Arterialised or Capillary.^a^	≤15 min	≥240 min
GIP [155]	Arterialised, Capillary, or Mixed Venous.^a^	≤15 min	≥240 min
GLP-1 [155]	Arterialised or Capillary.	≤15 min	≥240 min
Leptin^b^ [155]	Arterialised, Capillary, or Mixed Venous.^a^	≤240 min	≥360 min
PYY [156]	Arterialised or Capillary.^a^	≤15 min	≥360 min

*CCK* cholecystokinin, *GIP* glucose-dependent insulinotropic polypeptide, *GLP-1* glucagon-like peptide-1, *PYY* peptide-YY.

^a^Lack of evidence for a clear recommendation.

^b^Leptin often only measured in the fasted state.

### Posture

Changing the volume of distribution may affect the measured concentration even if the absolute amount of the analyte has not changed. Changing posture (e.g., supine versus sitting versus standing), impacts plasma volume and thus the concentrations measured. For example, changing posture from standing to supine increases plasma volume by >400 mL within 30 min, and return to standing decreases plasma volume by >500 mL within 30 min [92]. It is therefore recommended that posture is controlled (with a stabilisation phase of at least 30 min before sampling), or that plasma volume is measured to correct for changes in the volume of distribution.

## Appetite-related peptides

**Fig. 4:**
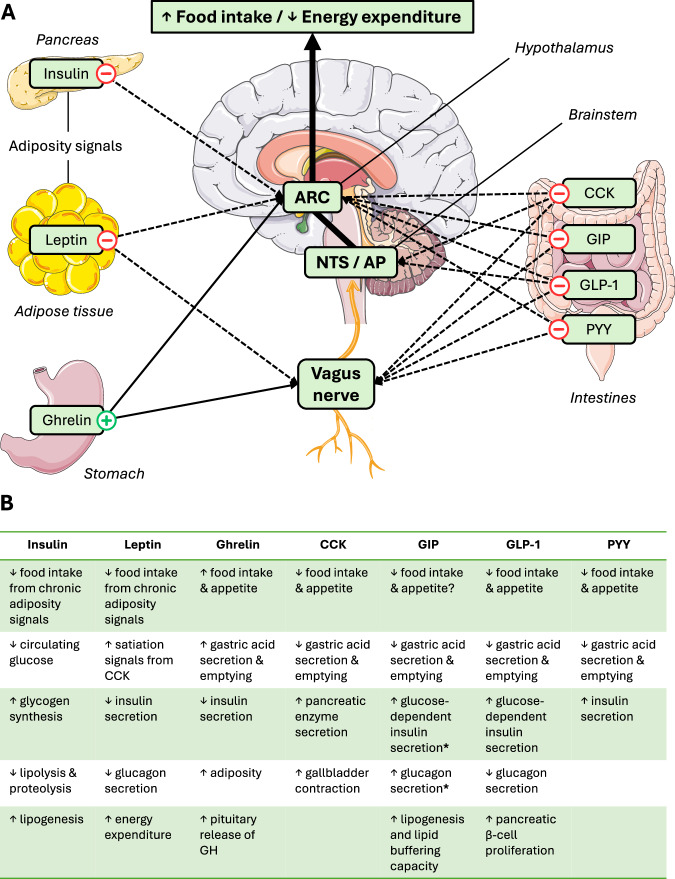
Overview of the role of appetite-related peptides in the neurohumoral control of food intake and energy expenditure. **A** Central and peripheral pathways involved in the appetite-related peptide control of food intake and energy expenditure. **B** Physiological functions of different appetite-related peptides. (+, solid line) indicates a positive effect on increasing food intake and/or decreasing energy expenditure; (−,dashed line) indicates a negative effect. * Indicates these effects may be blunted in conditions of hyperglycaemia. AP area postrema, ARC arcuate nucleus, CCK cholecystokinin, GH growth hormone, GIP gastric inhibitory polypeptide, GLP-1 glucagon-like peptide-1, NTS nucleus tractus solitarius, PYY peptide YY.

Appetite-related peptides communicate acute and chronic energetic/nutritional status to the central nervous system (Fig. 4). These neurohumoral messengers (peptides) include leptin—a tonic signal—which informs the brain about energy balance and available energy reserves. Concomitantly, ghrelin, cholecystokinin (CCK), GIP, GLP-1 and PYY primarily function in an episodic nature; influencing appetite sensations, eating behaviour and digestive processes in response to individual meals.

Certain appetite-related peptides are prone to degradation (in vivo and ex vivo) and circulate as different isoforms with divergent effects. Researchers must therefore consider which peptides to measure and ensure that blood sampling procedures maintain peptide integrity. Generic recommendations are listed in Table 4. Bespoke considerations for measuring episodic peptides are discussed herein. Tonic adiposity-related peptides, such as leptin and insulin, are less challenging to measure and are not addressed here. Likewise, glucagon [93] and CCK [94] measurements have been reviewed in detail elsewhere.

**Table 4:** Generic blood sampling and processing considerations prior to measurement of appetite-related peptides.

Recommendation	Explanation
Standardise participants’ body position for all blood draws (at least 20 min before each collection)	Body posture influences blood and plasma volume. The concentration of analytes in circulation may therefore vary if blood is drawn in different body positions (e.g. seated versus supine).
Check assay manufacturer guidelines when choosing blood sample collection tubes (e.g. EDTA, lithium heparin, clotting activator).	Peptide integrity is better preserved with some sample preservatives than others. Some preservatives interfere with assay biochemistry.
Pre-cool blood sample collection tubes by placing in wet ice prior to blood draws.	Ex vivo sample degradation is reduced at lower temperatures.
Use best practice phlebotomy techniques to avoid blood sample haemolysis (i.e. avoid excessively tight tourniquet and release immediately as blood flows, handle samples carefully, prompt centrifugation).	Haemolysis of blood samples may interfere in assay biochemistry and lead to missing data.
Centrifuge samples immediately after collection. If not possible, place samples in wet ice and process within 15 min.	Ex vivo sample degradation is reduced once plasma/serum is harvested.
Prepare single use sample aliquots to avoid assaying peptides after freeze-thaw cycles.	The freeze-thaw process may damage protein structure (particularly repeated freeze-thaws).
For long-term storage, freeze sample aliquots at −80 °C.	Colder sample storage (i.e. versus −20 °C) is likely to better preserve protein integrity.
Analyse samples in duplicate.	Duplicate analyses help to enhance result precision.
Analyse all samples from the same participant on the same assay plate/run. Within a research study, ensure all kits are from the same assay batch number where possible.	Inter-assay co-efficient of variation is greater than intra-assay co-efficient of variation.
Check the dynamic/working range when choosing appetite-related hormone assays.	Absolute concentrations of peptides may vary considerably depending on factors such as participant feeding status (fed versus fasted) and adiposity (obesity versus healthy weight).

### Ghrelin

Ghrelin is a 28 amino acid peptide most prominently secreted from the stomach. It is unique in being O-acylated at its third residue (serine) [95]. Ghrelin O-acyltransferase catalyses post-translational ghrelin acylation, permitting receptor binding and appetite effects [96]. Therefore, ghrelin circulates as acylated (AG) and unacylated (UAG) peptides. As UAG has diverging effects to AG, measurement of AG is recommended in appetite research. During blood sampling, this necessitates preservation of the octanoyl moiety which is prone to protease degradation [97].

Studies have investigated how sample processing affects circulating AG [18, 98–101]. Recommendations include the necessity to keep samples chilled; with pre-cooling of blood collection tubes and refrigerated centrifugation [101]. Samples should be centrifuged immediately or placed on ice for no longer than 15 min [99]. Protease inhibitor(s) must be added to samples; ideally to blood collection tubes to ensure immediate mixing [98, 99, 101]. Protease inhibitor choice should be based on assay recommendations. Sample acidification (hydrochloric acid) may be recommended for some assays to enhance preservation with long-term storage and freeze-thaw cycles [100].

### Incretins: GIP and GLP-1

GIP is a 42 amino acid peptide primarily secreted by the small intestine in its intact isoform, GIP_1-42_ [102]. Upon secretion, it undergoes rapid degradation ( ~ 5–7 min half-life) by dipeptidyl peptidase-4 (DPP-4) to form the inactive metabolite GIP_3-42_ [103], the predominant circulating isoform. In applied studies where GIP secretory responses to different interventions are of interest, it is recommended that researchers measure ‘total GIP’ [104]. However, GIP may act solely in an endocrine fashion, therefore researchers must consider whether the intact isoform alone may be of interest [104].

GLP-1 is primarily secreted from the intestine as two ‘intact’ isoforms: glycine-extended GLP-1_7-37_ and amidated GLP-1_7-36_ amide [102]. Like GIP, degradation by DPP-4 occurs ( ~ 1–2 min half-life) to form the primary metabolites, GLP-1_9-37_ and GLP-1_9-36_ amide [105, 106]. In humans, the amidated isoforms predominate [105, 107]. Only 10% of secreted GLP-1 reaches the circulation intact [106]; therefore, unlike GIP, intact GLP-1’s effects on satiety are predominantly through vagal afferents before degradation and systemic release [108]. Whilst the GLP-1 metabolites were initially thought to be inactive, this notion has been contested [109]. Given that the human intestine secretes 100% of GLP-1 in its intact form [110], and its rapid degradation alters concentrations between the secretion and measurement site (when sampling venous blood), peripheral concentrations of intact GLP-1 may not rise postprandially despite significant secretion [111]. It is similarly recommended to measure total GLP-1 (i.e. both GLP-1_7-36_ amide and GLP-1_9-36_ amide) when estimating GLP-1 secretory responses [104]. Conversely, measurement of intact GLP-1 alone may be relevant to specific scenarios, such as assessing responses to different DPP-4 inhibitors [104].

When measuring intact GIP and GLP-1, addition of a DPP-4 inhibitor to collection tubes before sample acquisition helps prevent ex vivo degradation [112]. Furthermore, ethanol or solid-phase extraction procedures can reduce non-specific interference by other plasma proteins [113]. These result in lower circulating concentrations and accentuate postprandial responses, enabling greater reproducibility across individuals, assays and studies [113]. These procedures are not necessary when measuring total GIP and GLP-1 [104]. Traditionally, aprotinin and blood collection tubes containing protease inhibitor cocktails (e.g. BD™ P800) have been used to further prevent degradation; however, recent investigations concluded EDTA-coated tubes without protease inhibitors are sufficient for clinical trials [114]. Immediate sample processing may obviate the need for these protease inhibitors.

Commercially available GLP-1 enzyme-linked immunosorbent assays (ELISA) were recently compared to an established in-house RIA [115]. Considerable variation in assay performance was observed between different assays and batches of the same assay. Therefore, analysing samples within the same batch using a highly specific and sensitive assay for the isoform(s) of interest is important [107]. Whilst radio-immunoassays were traditionally used, adequate ELISAs for intact and total GIP are commercially available [114]. Nevertheless, researchers should note that a short-form of GIP has recently been identified (GIP_1-30_ amide) which may possess similar biological activity to full-length GIP but is not detected by many current assays [112]. This could potentially underestimate GIP responses, however, specific assays for this isoform are under development [112].

### PYY

PYY is a 36 amino acid peptide secreted most prominently from L-cells in the distal intestine. In circulation, PYY exists in two major forms, as full-length PYY_1-36_ and as a 34 amino acid peptide (PYY_3-36_) [116]. The truncated peptide is produced by enzymatic cleavage of two amino-terminal peptides, by DPP-4 [117]. This step is crucial as only PYY_3-36_ can bind to the neuropeptide-Y (NPY) Y2-receptor, mediating appetite [97]. Sample collection and processing procedures must therefore inhibit the ex vivo conversion of PYY_1-36_ to PYY_3-36_ and further protease degradation.

Assay manufacturers typically recommend that samples destined for PYY_3-36_ analysis are treated with DPP-4 inhibitor and aprotinin (broad spectrum protease inhibitor). In experiments, blood is drawn into syringes containing DPP-4 inhibitor to block peptide truncation immediately, with aprotinin subsequently added. However, one study found that neither DPP4 inhibitor or aprotinin influenced fasted, fed, or postprandial concentrations of PYY_3-36_ [18]. The immediacy of sample processing (centrifugation) may explain this outcome and it is unclear how repeated freeze/thaw cycles would have impacted findings. EDTA and aprotinin treatment do, however, inhibit the degradation of PYY_3-36_ to PYY_3-34_ (inactive metabolite) [118]. If PYY_3-36_ cannot be measured, PYY_1-36_ and PYY_3-36_ broadly track one another from the fasted to postprandial state, meaning circulating levels of total PYY provide a reasonable marker of overall PYY secretion. However, there are scenarios where the ratio of PYY_1-36_ and PYY_3-36_ is altered [119]; therefore, assays directed at PYY_3-36_ should be prioritised when accurate quantification of the appetite-related isoform is the aim.

## Data handling

### Data presentation

Data from the MM-TT are characterised by the appetite-related outcome(s) quantified before and at multiple time-points after the meal stimulus. Appetite data are usually collected from two or more MM-TTs, showing data from participants studied under different conditions, different points in time and/or different populations.

**Fig. 5:**
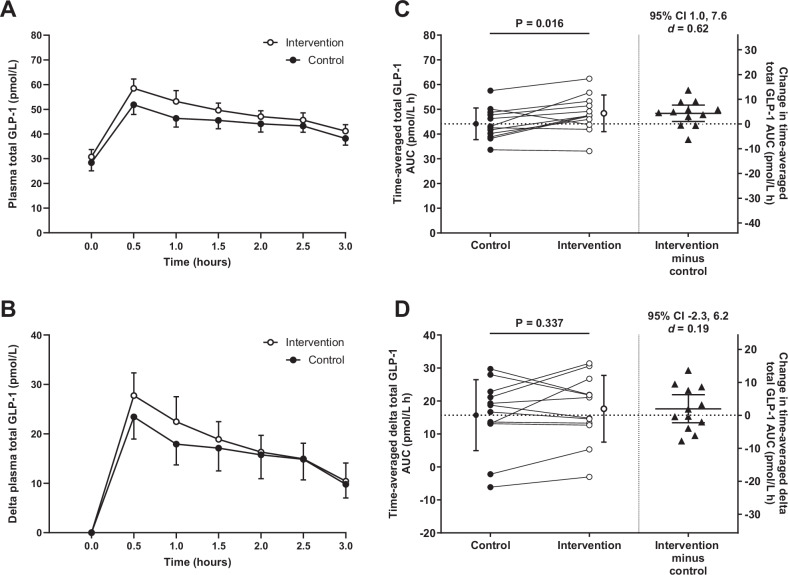
Plasma glucagon-like peptide-1 (GLP-1) concentrations during a 3-h mixed meal tolerance test with the meal provided after the fasted sample at 0-h. Data are hypothetical for *n* = 12 in a randomised crossover study involving an intervention and control study arm. On left hand panels, data presented as **A** raw analysed concentrations or **B** change from baseline (delta) and displayed as mean (SEM) to prevent figure distortion. On right hand panels **C**, **D**, the left hand *y* axis displays the mean (SD) time-averaged total area under the curve (AUC) and circles with connecting lines show the individual participant values. The right hand *y* axis displays the mean difference (95% confidence interval) between the intervention and control arms and the triangles represent the individual participant data. The *p* value, 95% confidence interval and standardised effect size (Cohen’s *d*) is presented for the mean difference between the intervention and control study arms.

Variables with multiple postprandial assessments are often presented graphically as a response curve over time (Fig. 5A). As an alternative to raw values, appetite perceptions and appetite-related hormone concentrations can be presented and analysed as changes from baseline (Fig. 5B and Table 2). This may minimise the influence of day-to-day variability and improve sensitivity to examine predictors of interest [120]. For outcomes limited to single or possibly duplicate assessments, or those converted to summary values (see below), raw data may be better suited to presentation in table format or in visual plots that display the data distribution [121, 122] (Fig. 5C, D).

### Data analysis

Raw data from every sampling time-point during the MM-TT can be integrated into a statistical model as an independent variable with other predictors such as condition or group. This allows examination of the mean effect of each independent variable on the appetite outcome, averaging across the levels of the other independent variables. The main effect of time can identify differences in the time course of responses across the level of another independent variable when integrated into an interaction term.

Researchers are often less concerned with the influence of sampling time so it may be preferable to remove the time series element by calculating a summary statistic. This approach allows for direct comparisons of summary condition or group means, with adjustment for baseline (pre-intervention) as a covariate recommended for longitudinal study designs [123, 124]. Temporal data can be condensed into a summary statistic by calculating the area under the curve (AUC) using the trapezoidal rule. The total AUC involves summing the AUC calculated between the origin line at zero and the response curve for each consecutive sampling point, generating a single value representing a time-weighted average of the postprandial response [125]. This can be converted to a time-averaged value by dividing by the observation period which is often easier to interpret (Fig. 5C).

Whilst the total AUC indicates the overall response, it does not adjust for baseline variability (unless calculated using delta values [Fig. 5D]). Alternatively, the incremental AUC can be quantified using the same method as the total AUC but is restricted to areas above a nominal threshold whilst ignoring areas below the threshold [126, 127]. The threshold adopted is commonly the fasting value, but for outcomes such as acylated ghrelin that decrease in response to meals, the nadir value may be more appropriate. For the MM-TT, appetite responses to the meal are the primary focus and since baseline (fasting) values can vary, incremental AUC may be preferred over total AUC to better isolate meal-induced effects [128]. The freely available *Time Series Response Analyser* provides an automated tool for the accurate calculation of several summary statistics [128]. In addition to AUC, peak or nadir and time-to-peak or time-to-nadir values can be calculated for each response curve providing sufficient sampling time-points are collected to generate precise estimates.

### Data considerations—sample size and target difference

No clinically relevant target differences have been established for appetite parameters. Establishing an important difference is central to sample size estimations and interpreting study findings and can be guided by relevant literature [129]. For example, a 10-mm (10%) difference in mean or AUC appetite has been proposed as a realistic difference between two meals [23]. Difference estimates can be supplemented by standardised effect sizes often expressed for continuous outcomes using Cohen’s *d* thresholds (0.2, 0.5 and 0.8 for small, medium, and large effects, respectively) [130]. Clinical or practical relevance of MM-TT findings should not depend solely on statistical significance but also be informed by the target difference and precision of treatment estimates reflected in confidence intervals [129, 131].

### Data considerations—missing data

Missing MM-TT data is almost inevitable when multiple time-sensitive measures are collected on repeat occasions. Comprehensive guidelines exist for handling missing data in randomised controlled trials [132–134] and many of these principles hold for time series data collected in MM-TTs. Single imputation methods using the sample mean or regression-based predictions may be appropriate when missingness is completely random (e.g., a missing blood sample due to sampling issues). A tool has been developed to simulate missing MM-TT data that accounts for the correlated nature of time series data and could be useful when ≤2 consecutive values are missing [135]. Other approaches may be required if missing data can be explained by observed variables (e.g., greater missing samples in women than men) or if missingness is systematically dependent on unobserved data (e.g., less follow-up data in those benefiting less from an intervention) [132]. These include multiple imputation and mixed effects models which can be supplemented by sensitivity analyses to assess the impact of missing data on treatment estimates [132].

### Data considerations—outliers

Outliers can be identified visually from boxplots, histograms, or scatterplots, and/or mathematically using the mean or median. For example, standardised residuals, calculated by dividing the difference between the observed and mean predicted value by its standard deviation, beyond a threshold of 3.5 has been proposed for sample sizes <50, >4 for samples up to 500, >4.5 for samples up to 5000, and so forth [131]. Alternatively, a threshold of the median ±2.5 times the median deviation of scores from the median is likely to be less sensitive to the influence of outliers than calculation of the mean and standard deviation [136]. Outliers should not be excluded automatically without strong evidence that the outlier is not representative of the population of interest. Findings should be presented with and without the outlier(s) included and with full transparency on the process adopted for outlier identification [137].

### Inter-individual variability

Although most studies quantify mean treatment or group effects, inter-individual variability may exist in appetite responses to identical meals beyond within-subject random variation [138]. Inter-individual variability in the magnitude of postprandial appetite suppression is an important consideration but requires robust methods to quantify. This can be established by replication of treatment and control arms (replicate crossover design) allowing separation of individual response heterogeneity from measurement error and/or natural fluctuation in outcomes over time [139, 140].

## Concluding remarks and development of the MM-TT checklist

This paper has sought to highlight the plethora of factors that require consideration when designing MM-TTs. We hope this paper can be used as a resource by researchers working in relevant fields, helping to support decisions about MM-TT design in the planning stages of research. While the primary focus of this paper is the appetite research field, various elements are relevant to those using MM-TTs in other research contexts. By scrutinising all elements of the MM-TT, this paper should help researchers to better understand the MM-TT process including the ability to recognise strengths and limitations in its application. To support these ambitions, we have developed a checklist (Supplementary Table 1**)** which pools key factors to consider when designing a MM-TT. Researchers can use this list to evaluate their choice of methods and we encourage its adaptation for use in wider fields of research.

It is important to note that this paper did not seek to identify a ‘gold standard’ MM-TT to be recommended for all. This is because the MM-TT technique is used in a range of contexts, with varied research aims and available resources. Instead, our review has highlighted key factors which underpin the adaptability of this method. Also relevant to note, in some cases we have been unable to make clear recommendations given lack of evidence. There are many examples of this regarding familiarisation and standardisation procedures, for instance. Moreover, for many issues the methodological approach depends on the research question. Finally, the scope of this paper was limited to core MM-TT factors. Further discussion about wider, related constructs and techniques (e.g. measurement of energy intake, diet-induced thermogenesis, gastric emptying) is needed in future work.

## Supplementary information

Supplementary Table 1

## Data Availability

This review paper does not contain any original data. All information presented is derived from previously published studies, which are cited appropriately within the text. As such, no new data were generated or analysed during the preparation of this manuscript.
