# Supplementary material for: The mixed-meal tolerance test as an appetite assay: methodological and practical considerations
Source: Int J Obes (Lond). 2025 Jul 30;49(11):2168–83. doi: 10.1038/s41366-025-01866-7 (PMC12583139; doi:10.1038/s41366-025-01866-7)
Supplement: Supplementary file 1 — Supplementary Table 1 [file 41366_2025_1866_MOESM1_ESM.docx]

**Mixed-Meal Tolerance Test Protocol Design Checklist**

This checklist can be used by researchers when planning a mixed-meal tolerance test (MM-TT) to support their decisions regarding MM-TT design.

| Consideration | Description | ☑/⮽ |
| --- | --- | --- |
| Participant familiarisation | | |
| Research environment | Participants have seen and/or spent time in the facilities where the MM-TT is due to take place. |  |
| Blood sampling procedures | Phlebotomy procedures have been explained to participants. This may include participants being shown needles and blood collection tubes. In some instances, a ‘dummy’ intravenous cannulation may be performed. |  |
| Meal test foods | Participants have confirmed that they like or are able to eat all test foods. Participants may be given an opportunity to consume test meals before main trials. |  |
| Questionnaires / visual analogues scales | Participants should understand any nuances regarding the completion of questionnaires during a MM-TT. Participants should be given an opportunity to complete a visual analogue scale before main trials. |  |
| Standardisation of methods & techniques | | |
| Test meal presentation (physical appearance) | Test meals should be presented to all participants in a consistent manner. This relates to the use of crockery, eating utensils and the meal appearance e.g. slicing and stacking of foods. |  |
| Test meal presentation (verbal instructions) | Researchers verbal instructions to participants when presenting the test meal should be identical each time. |  |
| Extraneous food cues in the laboratory | Factors which may influence appetite and eating behaviour must be controlled within the laboratory. This includes access to videos, television, internet, magazines, smells, conversations with/between experimenters. |  |
| Laboratory environmental conditions | The temperature and humidity of research laboratories should be standardised and monitored throughout. |  |
| MM-TT start time | MM-TTs should start at the same time of day both within participants (if more than one is completed i.e. pre to post intervention) and between participants. If a MM-TT occurs in the morning following an overnight fast, consideration should be given to when the final pre-test meal is consumed. |  |
| Duration for test meal consumption | Time taken to consume the test meal should be similar within and between participants. This may require some instruction from researchers and observation (covert) to confirm adherence. |  |
| Dietary intake | Participants should maintain a typical diet in the days before a MM-TT (at least 48 h) i.e. not engage in any extreme dietary manipulation. If participants are completing more than one MM-TT (e.g. intervention studies) then standardisation can be facilitated with weighed food records or through research teams providing all foods and drinks to be consumed in the standardisation period. |  |
| Physical activity | In the 48 h before an MM-TT, participants should either refrain from structured exercise or record what they do and replicate their activity ahead of subsequent trials. Accelerometry can be used to confirm adherence to instructions. During the measurement period after test meal consumption, researchers may consider standardising and/or limiting physical activity within the laboratory e.g. participants remain seated. |  |
| Alcohol | Alcohol consumption should be prohibited within the 48 h before a MM-TT. |  |
| Caffeine | High caffeine-containing products may be prohibited in the 48 h before MM-TTs, or use may be moderated. Recording and replication of intake is necessary if participants complete more than one MM-TT (e.g. intervention studies). |  |
| Hydration / water provision | Participants should consume approximately 500 mL of water on waking if MM-TTs are undertaken in the morning (if not, in the hours before the MM-TT). Participants should refrain from consuming larger amounts of water in the hour before the MM-TT begins. Water consumption during a MM-TT should be moderated, recorded, and replicated if participants complete more than one test. |  |
| Sleep | Participants should maintain a healthy habitual sleep routine in the days before a MM-TT (7 to 9 h). On the night before an MM-TT, participants’ bedtime and wake time should be standardised within intervention studies. A sleep diary and/or accelerometer capable of measuring sleep parameters may facilitate adherence. |  |
| Smoking / vaping / e-cigarettes | Smoking, vaping and use of e-cigarettes should be standardised on the day before MM-TTs and also in the hours before the test. These products should not be used during a MM-TT. If participants are completing more than one MM-TT (e.g. intervention study), use should be replicated ahead of subsequent assessments. |  |
| Menstrual cycle and oral contraceptives | For studies making within participant and between participant comparisons, all females should be tested in the same phase of the menstrual cycle. Verification of menstrual cycle phase should be made through measurements of circulating sex-hormones, or via self-report if not feasible. Additional considerations should be made regarding the inclusion or exclusion of oral contraceptive use. Regardless, the oral conceptive use status of the participant should remain consistent throughout the duration of the study. |  |
| Test meal | | |
| Fixed vs. scaled meal | Consideration of whether the energy content of the test meal is identical for all participants or scaled based on a participant-level factor i.e. body weight, lean mass, resting metabolic rate, total daily energy expenditure. |  |
| Macronutrient composition | Consider whether a balanced test meal is provided (e.g. 50% carbohydrate, 35% fat, 15% protein) or if there is a reason for providing a meal with a different macronutrient composition. Consideration should be given to the quality of macronutrients within the meal i.e. types of carbohydrate, fat and protein. |  |
| Physical state | Is the test meal composed of whole foods or an oral nutritional (liquid) supplement? |  |
| Blood sampling | | |
| Sampling location | Consideration of whether arterial, arterialised, venous or capillary blood samples are collected. |  |
| Sampling frequency | Consideration of how many blood samples are collected during the MM-TT and the interval between each sample. |  |
| Posture | All blood samples for an individual participant should be collected in the same position (e.g. supine, semi-supine). |  |
| Timing | Blood samples should be collected after appetite data are obtained i.e. VAS. |  |
| Appetite-related peptides | | |
| Peptide choice | Consideration of which appetite-related peptides should be measured to address the research question. |  |
| Peptide isoform | Of the peptides to be measured, which form of the individual peptides should be measured. |  |
| Sample handling | Sample collection and handling processes must preserve peptide integrity. Researchers must consider factors including sample collection tubes, sample treatments (i.e. protease inhibitors), transit time/temporary ice storage, centrifuge routine (temperature, RCF, duration). |  |
| Sample storage | For long-term storage of samples, considerations include the freezer temperature (20 vs. 80 degrees centigrade), storage duration, and the number of aliquots stored. The latter may impact the number of freeze-thaw cycles the samples are exposed to which should be minimised. |  |
| Sample analysis | Considerations regarding assay choice including RIA vs. ELISA, dynamic range, intra- and inter-assay coefficient of variation (CV%). |  |
| Data handling | | |
| Data presentation | Consideration of how data will be presented i.e. graph, table or text. |  |
| Data analysis | Consideration of the statistical approach. For instance, will raw data be analysed or will analysis be based on summary variables (e.g. AUC). Which statistical tests will be used. Will models adjust for covariates and/or assess delta values (change from baseline). This information is needed *a priori* to inform sample size calculations and should be documented in trial registries. |  |
| Data considerations (sample size / target difference) | Identification of clinically relevant or meaningful effects relevant to the research question and outcomes. This may be based on raw outcome data or effect sizes. |  |
| Data considerations (missing data) | Documentation of the method for how missing data will be dealt with before analysis. |  |
| Data considerations (outliers) | Documentation of the method for how outliers are identified within datasets. |  |
